# Resistance of Asian *Cryptococcus neoformans* Serotype A Is Confined to Few Microsatellite Genotypes

**DOI:** 10.1371/journal.pone.0032868

**Published:** 2012-03-13

**Authors:** Weihua Pan, Kantarawee Khayhan, Ferry Hagen, Retno Wahyuningsih, Arunaloke Chakrabarti, Anuradha Chowdhary, Reiko Ikeda, Saad J. Taj-Aldeen, Ziauddin Khan, Darma Imran, Ridhawati Sjam, Pojana Sriburee, Wanqing Liao, Kunyaluk Chaicumpar, Natnicha Ingviya, Johan W. Mouton, Ilse Curfs-Breuker, Teun Boekhout, Jacques F. Meis, Corné H. W. Klaassen

**Affiliations:** 1 Shanghai Key Laboratory of Molecular Medical Mycology, Institute of Dermatology and Medical Mycology, Changzheng Hospital, Second Military Medical University, Shanghai, People's Republic of China; 2 CBS-KNAW Fungal Biodiversity Centre, Department of Yeast and Basidiomycete Research, Utrecht, The Netherlands; 3 University Medical Center Utrecht, Department of Internal Medicine and Infectious Diseases, Eijkman Winkler Institute, Utrecht, The Netherlands; 4 Department of Microbiology and Parasitology, Faculty of Medical Science, University of Phayao, Phayao, Thailand; 5 Division of Mycology, Department of Parasitology, Faculty of Medicine, University of Indonesia, Jakarta, Indonesia; 6 Department of Parasitology, Faculty of Medicine, Christian University of Indonesia, Jakarta, Indonesia; 7 Department of Medical Microbiology, Postgraduate Institute of Medical Education and Research, Chandigarh, India; 8 Department of Medical Mycology, Vallabhbhai Patel Chest Institute, University of Delhi, Delhi, India; 9 Department of Microbiology, Meiji Pharmaceutical University, Tokyo, Japan; 10 Mycology Unit, Microbiology Division, Department of Laboratory Medicine and Pathology, Hamad Medical Corporation, Doha, Qatar; 11 Department of Microbiology, Faculty of Medicine, Health Sciences Centre, Kuwait University, Jabriya, Kuwait; 12 Department of Neurology, Faculty of Medicine, University of Indonesia, Jakarta, Indonesia; 13 Department of Neurology Cipto Mangunkusumo Hospital, Jakarta, Indonesia; 14 Department of Microbiology, Faculty of Medicine, Chiang Mai University, Chiang Mai, Thailand; 15 Department of Microbiology, Faculty of Medicine, Khon Kaen University, Khon Kaen, Thailand; 16 Department of Pathology, Faculty of Medicine, Prince of Songkla University, Hat Yai, Thailand; 17 Department of Medical Microbiology and Infectious Diseases, Canisius Wilhelmina Hospital, Nijmegen, The Netherlands; 18 Department of Medical Microbiology, Radboud University Nijmegen Medical Center, Nijmegen, The Netherlands; Duke University Medical Center, United States of America

## Abstract

**Background:**

*Cryptococcus neoformans* is a pathogenic yeast that causes cryptococcosis, a life threatening disease. The prevalence of cryptococcosis in Asia has been rising after the onset of the AIDS epidemic and estimates indicate more than 120 cases per 1,000 HIV-infected individuals per year. Almost all cryptococcal disease cases in both immunocompromised and immunocompetent patients in Asia are caused by *C. neoformans* var. *grubii*. Epidemiological studies on *C. neoformans* in pan-Asia have not been reported. The present work studies the genetic diversity of the fungus by microsatellite typing and susceptibility analysis of approximately 500 isolates from seven Asian countries.

**Methodology/Principal Findings:**

Genetic diversity of Asian isolates of *C. neoformans* was determined using microsatellite analysis with nine microsatellite markers. The analysis revealed eight microsatellite complexes (MCs) which showed different distributions among geographically defined populations. A correlation between MCs and HIV-status was observed. Microsatellite complex 2 was mainly associated with isolates from HIV-negative patients, whereas MC8 was associated with those from HIV-positive patients. Most isolates were susceptible to amphotericin B, itraconazole, voriconazole, posaconazole, and isavuconazole, but 17 (3.4%) and 10 (2%) were found to be resistant to 5-flucytosine and fluconazole, respectively. Importantly, five Indonesian isolates (approximately 12.5% from all Indonesian isolates investigated and 1% from the total studied isolates) were resistant to both antifungals. The majority of 5-flucytosine resistant isolates belonged to MC17.

**Conclusions:**

The findings showed a different distribution of genotypes of *C. neoformans* var. *grubii* isolates from various countries in Asia, as well as a correlation of the microsatellite genotypes with the original source of the strains and resistance to 5-flucytosine.

## Introduction

*Cryptococcus neoformans* and *C. gattii* are encapsulated basidiomycetous yeasts that can cause life-threatening infections in humans. According to the current classification, *C. neoformans* consists of two varieties, namely variety *grubii* (serotype A) and variety *neoformans* (serotype D). *C. neoformans* and *C. gattii* differ in ecology, biochemistry, molecular characteristics, and their ability to cause disease [1], [2]. *C. neoformans* is known as an opportunistic pathogen because it mainly infects immunocompromised patients, whereas *C. gattii* is considered a primary pathogen that infects otherwise healthy individuals [3]. Clinical characteristics of *C. neoformans* and *C. gattii* differ as well. The latter species causes more frequently cryptococcomas and has a lower susceptibility to antifungal agents, resulting in prolonged treatment and a higher mortality rate when compared to *C. neoformans* [4].

*Cryptococcus neoformans* continues to be the most important cause of fungal meningitis in immunocompromised patients. The global burden of cryptococcal infections among HIV-infected patients is estimated at nearly one million new cases per year [5]. In South Asia and Southeast Asia, the incidence of HIV infection is the second-highest with over 4.5 million HIV-infected patients. The prevalence of cryptococcal meningitis was estimated to be 13.6 and 120 per thousand HIV-infected individuals per year among HIV-infected patients in these two regions, respectively [5]. In contrast, most cases of cryptococcosis in East Asia, especially in China and Japan, where HIV prevalence is low, have been reported from apparently immuncompetent patients and were mostly caused by *C. neoformans* var. *grubii* [6]–[9]. Similarly, Vietnamese patients with cryptococcal meningitis were usually infected by this variety, but here it manifests in both immunocompromised and immunocompetent patients [10].

Most cryptococcal meningitis cases in Asia are caused by *C. neoformans* [7]. Notwithstanding the clinical importance of the fungus in this part of the world, a systematic survey on the genetic diversity of the pathogen has not been performed. Genotyping of isolates may reveal differences in host range and clinical symptoms. Recently, a genotyping study of Vietnamese clinical isolates using amplified fragment length polymorphism (AFLP) revealed two genotypes, VNIγ and VNIδ with the former as the major genotype for isolates originating from non-HIV-infected patients [10]. For epidemiological studies of the *C. neoformans*/*C. gattii* complex, several molecular methods have been used, such as AFLP, M13-based PCR fingerprinting, Multi-locus Sequence Typing (MLST) and analysis of the intergenic spacer (IGS) ribosomal DNA sequences [11]–[15].

Microsatellite analysis is a genotyping technique that is becoming increasingly popular for molecular typing of medically important fungi [16]–[18]. Microsatellites, also referred to as short tandem repeats (STRs), are genomic sequences that consist of tandem repeated short motifs [19]. Mutations in microsatellites lead to a change in the number of repeats creating genotypic diversity. In some fungi, e.g. *Aspergillus fumigatus* and *A. flavus*, molecular typing using microsatellites proved to be more discriminatory than MLST [18], [19]. In our study, microsatellite typing was applied to estimate the extent of genetic diversity and the epidemiological relationships of a collection of clinical cryptococcal isolates that originated from East, South and Southeast Asia, and the Middle East. Moreover, a set of environmental isolates from Japan and Thailand was included. *In vitro* antifungal susceptibility was determined for amphotericin B, flucytosine, fluconazole, itraconazole, voriconazole, posaconazole and isavuconazole. The aims of this study were: (i) to analyze the genotypic diversity as well as the distribution of *C. neoformans* var. *grubii* from different geographical regions in Asia, (ii) to relate the genetic background of the cryptococcal isolates to disease status and origin from the human body, (iii) to test the *in vitro* antifungal susceptiblity of the isolates against seven antifungal drugs, and (iv) to determine if differences in susceptibility correlate with the observed genotypic diversity.

## Materials and Methods

### Isolates and media

A total of 426 clinical isolates of *Cryptococcus neoformans* var. *grubii* isolates were obtained from the collections of the Chinese *Cryptococcus* Reference Centre at the Second Military Medical University, Shanghai, China (*n* = 115); Department of Microbiology, Faculty of Medicine, Chiang Mai University, Chiang Mai, Thailand (*n* = 79); Department of Medical Microbiology, Postgraduate Institute of Medical Education and Research, Chandigarh, India (*n* = 61); Sappasitthiprasong Hospital, Ubon Ratchathani, Thailand (*n* = 59); Department of Parasitology, Faculty of Medicine, University of Indonesia, Jakarta, Indonesia (*n* = 40); Department of Microbiology, Meiji Pharmaceutical University, Tokyo, Japan (*n* = 28); Department of Microbiology, Faculty of Medicine, Khon Kaen University, Khon Kaen, Thailand (*n* = 20); Department of Microbiology, Faculty of Medicine, Health Sciences Centre, Kuwait University, Jabriya, Kuwait (*n* = 10); Mycology Unit, Microbiology Division, Department of Laboratory Medicine and Pathology, Hamad Medical Corporation, Doha, Qatar (*n* = 5) and Department of Pathology, Faculty of Medicine, Prince of Songkla University, Hat Yai , Thailand (*n* = 9) (Table S1). Furthermore, 67 environmental isolates were obtained from the Department of Microbiology, Faculty of Medicine, Chiang Mai University, Thailand (*n* = 58) and the Department of Microbiology, Meiji Pharmaceutical University, Tokyo, Japan (*n* = 9) (Table S2). Two-hundred and thirty-six isolates originated from HIV-infected patients and 156 isolates were obtained from HIV-negative patients. Thirty-four out of 426 clinical isolates were from patients with unknown HIV status.

Species identification was initially performed by standard mycological methods [20]. l-Canavanine-glycine-bromothymol blue (CGB) medium was used to distinguish between *C. neoformans* and *C. gattii* isolates [21]. *Cryptococcus* isolates were stored in sterile 2 ml screw-capped tubes containing porous beads (Microbank, ProLab Diagnostics, Richmond Hill, ON, Canada) at −80°C until further use.

### Mating- and serotype analysis by PCR, and microsatellite typing

Genomic DNA extraction was performed as previously described [22]. All PCR amplifications for mating- and serotyping were carried out in a total volume of 20 µl containing 0.1 mM dNTPs, 0.5 U of Taq DNA polymerase (Gentaur, Brussels, Belgium), 1 µl of template genomic DNA (100 ng/µl) and 0.5 µM of the forward and reverse primers, as described by Bovers et al. [23] in 1× PCR reaction buffer containing 50 mM MgCl_2_ [24].

Microsatellite analysis was performed using nine microsatellite markers [17]. However, instead of three multiplex PCRs, each locus was amplified in a separate PCR reaction and reaction products containing different fluorescent labels were pooled prior to analysis. One microliter of combined reaction product was mixed with 8.75 µl water and 0.25 µl ET-550R ROX Size Standard (GE Healthcare, Diegem, Belgium). Samples were analyzed on a MegaBACE 500 automated DNA analysis platform (GE Healthcare) equipped with a 48 capillary array according to the instructions of the manufacturer. Repeat numbers were assigned using Fragment Profiler v1.2 (GE Healthcare), imported into BioNumerics v6.0 software (Applied Maths, Sint Martens-Latem, Belgium) and analyzed using the multistate categorical similarity coefficient. According to previously described criteria, microsatellite complexes (MCs) were defined as groups of two or more microsatellite genotypes that differ by a maximum of two loci [17].

### Antifungal susceptibility testing

*In vitro* antifungal susceptibility testing of amphotericin B (AMB; Bristol Myers Squibb, Woerden, The Netherlands), flucytosine (5FC; Valeant Pharmaceuticals, Zoetermeer, The Netherlands), fluconazole (FLU; Pfizer Central Research, Sandwich, Kent, United Kingdom), itraconazole (ITR; Janssen Cilag, Tilburg, The Netherlands), posaconazole (POS; Schering-Plough Corp., Kenilworth, NJ, USA), voriconazole (VOR; Pfizer Central Research) and isavuconazole (ISA; Basilea Pharmaceutica, Basel, Switzerland) was performed using the standard broth microdilution method as described in CLSI document M27-A3 [25]. The minimal inhibitory concentrations (MIC) were read optically after 72 h of incubation at 35°C. For AMB, the MIC was defined as the lowest concentration of drug showing no yeast growth. For the other antifungal compounds, the MIC was defined as the lowest concentration that caused a prominent reduction of yeast growth (≥50%). *Candida krusei* ATCC6258 and *Candida parapsilosis* ATCC22019 were used as quality controls.

The resistance breakpoints of FLU and 5FC were taken from CLSI document M27-A3 [25] and Perfect et al. [26] as follows: ≥16 µg/ml for FLU; ≥32 µg/ml for 5FC. According to Nguyen and Yu [27] the resistance breakpoint of AMB is ≥2 µg/ml. Interpretive criteria of ITR and the new azoles have not been proposed yet for *C. neoformans*. However, a MIC≤1 µg/ml was suggested as the susceptibility breakpoint for ITR, VOR, POS and ISA [28]–[31].

### Data Analysis

The Simpson's index of diversity was calculated to assess the genotypic diversity of microsatellite genotypes among the different populations [32]. The value obtained is scaled from zero to one, where a value of one indicates that all isolates are different, and a value of zero means that all belong to the same genotype.

Chi-square and Fisher exact tests were applied to examine the correlation between MCs and isolate categories, including HIV status, sample sources (environmental or clinical), and geographical origin. *In vitro* antifungal susceptibility testing results were statistically analyzed using the t-test method. *P* values less than 0.05 were considered significant for all statistical methods. The statistics were analyzed using StatsDirect v2.7.8 (StatsDirect, Cheshire, United Kingdom).

## Results

### Mating-type and serotype analysis

Of the 426 clinical *C. neoformans* var. *grubii* isolates obtained from HIV-positive patients (*n* = 236), HIV-negative patients (*n* = 156), and those with an unknown HIV status (*n* = 34), 425 isolates (99.8%) belonged to mating-type α and serotype A (αA), and one isolate (0.2%) from a HIV-negative patient in China belonged to mating-type α and serotype AD (αAαD). All 67 environmental isolates of *C. neoformans* var. *grubii* obtained from avian droppings from Chiang Mai, Thailand (*n* = 58) and Tokyo, Japan (*n* = 9) were mating type α and serotype A (Table S1 and Table S2).

### Microsatellite genotyping

**Figure 1:**
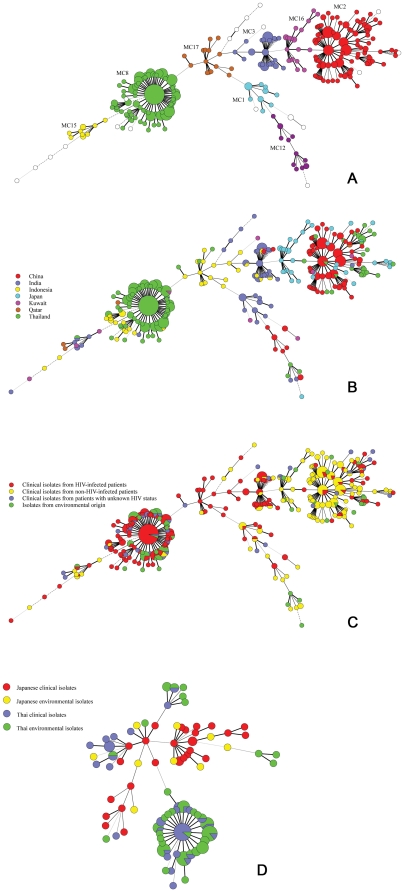
Genotypic variation of *C. neoformans* isolates from different Asian countries by microsatellite typing. (A) Minimum spanning tree based on a multistate categorical analysis representing 429 *C. neoformans* var. *grubii* isolates from different countries. Each circle represents a unique genotype. The size of the circle corresponds to the number of isolates within that genotype. Numbers and connecting lines correspond to the number of different markers between genotypes. Genotypes with identical colors are part of a microsatellite complex (MC). Circles without color are unique genotypes that are not part of a MC.; (B) Same as A, but now showing the genotypes from different geographic locations. Different colors correspond to different countries.; (C) Same as A and B, but now showing the genotypes from clinical and environmental sources.; (D) Same as A, B and C, but now showing the genotypes of Thai and Japanese population from clinical and environmental sources.

The genetic diversity of 492 isolates of *C. neoformans* var. *grubii* (αA) and one αAαD hybrid isolate was analyzed by microsatellite typing. Within this collection of 493 isolates, 265 different genotypes were found. These 265 genotypes were distributed over eight microsatellite complexes (MCs), which were defined as described previously [17]. Of the eight observed MCs, five were previously found in a collection of cryptococcal isolates from Cuba (i.e., MC1, MC2, MC3, MC8 and MC12), while three novel MCs were observed in the Asian collection presented here. These were assigned MC15, MC16, and MC17 (Figure 1A, Table S1 and Table S2). Twenty-four of the 493 isolates, which belonged to 19 microsatellite genotypes, could not be assigned to a MC. MC8 was the predominant MC among Asian isolates (*n* = 206, 41.8%), followed by MC2 (*n* = 151, 30.6%), MC3 (*n* = 42, 8.5%), MC16 (*n* = 21, 4.3%), MC17 (*n* = 15, 3.1%), MC1 (*n* = 14, 2.8%), MC12 (*n* = 13, 2.6%) and MC15 (*n* = 11, 2.2%). The distribution of MCs was found to differ in each country (Figures 1A and B, and Table 1). MC2 was predominant among the Chinese, Japanese, and Thai cryptococcal populations, whereas MC8 was the major type among isolates of patients from Southeast Asian countries (i.e., Indonesia and Thailand). In India, the majority of isolates belonged to MC1 and MC3. In addition, MC16 and MC17 were the predominant MCs containing Japanese and Indonesian isolates, respectively. The 17 isolates studied from the Middle East (Kuwait and Qatar) occur more scattered and belonged to MC2, MC3, MC8, and MC15 (Figures 1A and B, and Table 1). The relationship between most MCs is not well resolved. However, MC2 and MC16 that contained isolates from the Chinese, Thai, and Japanese populations are well interconnected.

**Table 1:** Distribution of microsatellite complexes (MCs) between different countries.

MCs	China*n* (%)	Japan*n* (%)	India*n* (%)	Indonesia*n* (%)	Thailand*n* (%)	Kuwait*n* (%)	Qatar*n* (%)	Total number of isolates*n* (%)
MC1	0	0	**14 (23)**	0	0	0	0	14 (2.8)
MC2	**102 (88.7)**	**14 (37.8)**	1 (1.6)	0	**30 (13.3)**	2 (20)	2 (40)	151 (30.6)
MC3	2 (1.7)	0	**30 (49.2)**	9 (22.5)	0	1 (10)	0	42 (8.5)
MC8	0	0	3 (4.9)	**17 (42.5)**	**183 (81.3)**	2 (20)	1 (20)	206 (41.8)
MC12	8 (7)	0	2 (3.3)	0	3 (1.3)	0	0	13 (2.6)
MC15	0	0	6 (9.8)	0	1 (0.5)	2 (20)	2 (40)	11 (2.2)
MC16	0	**20 (54.1)**	0	0	1 (0.5)	0	0	21 (4.3)
MC17	0	0	0	**12 (30)**	3 (1.3)	0	0	15 (3.1)
None MCs	3 (2.6)	3 (8.1)	5 (8.2)	2 (5)	4 (1.8)	3 (30)	0	20 (4.1)
Total	115	37	61	40	225	10	5	493

The predominant MCs in each country are indicated in bold.

The genotypic diversity of *C. neoformans* var. *grubii* was found to be most diverse (*D* = 1.000) in the Kuwait and Qatar populations, followed by the Japanese (*D* = 0.998), Indonesian (*D* = 0.994), Indian (*D* = 0.983), Chinese (*D* = 0.975), and Thai populations (*D* = 0.968) (Table 2). The genetic diversity within MCs showed values of Simpson's index of diversity close to 1, thus indicating that each MC contained a high level of genetic diversity. A significant correlation between MCs and HIV-status was observed by the Chi-square (*p*<0.0001) and Fisher exact tests (*p*<0.0001) (Figures 1A and C, and Table 3). The majority of isolates from HIV-negative patients belonged to MC2 that accounted for 104 out of 156 isolates from HIV-negative patients (66.6%), followed by MC16 (*n* = 13, 8.3%). In contrast, MC8 was the predominant MC containing isolates from HIV-positive patients (138 of 236, 58.5%), followed by MC2 (*n* = 30, 12.7%), MC3 (*n* = 33, 14%) and MC17 (*n* = 13, 5.5%).

**Table 2:** Simpson's index of diversity (*D*) in geographically different populations and for different microsatellite complexes (MCs).

Country	*D_Country_*	MCs	*D_MCs_*
China	0.975	MC1	0.912
Japan	0.998	MC2	0.983
India	0.983	MC3	0.945
Indonesia	0.994	MC8	0.960
Thailand	0.968	MC12	0.974
Kuwait	1.000	MC15	0.964
Qatar	1.000	MC16	0.990
		MC17	1.000

**Table 3:** Distribution of microsatellite complexes (MCs) from patients according to HIV status.

MCs	HIV positive*n* (%)	HIV negative*n* (%)	Unknown status*n* (%)	Total number of isolates*n* (%)
MC1	6 (2.5)	6 (3.9)	2 (5.9)	14 (3.3)
MC2	30 (12.7)	**104 (66.6)**	6 (17.7)	140 (32.9)
MC3	33 (14)	8 (5.1)	1 (2.9)	42 (9.9)
MC8	**138 (58.5)**	4 (2.6)	19 (55.9)	161 (37.8)
MC12	2 (0.9)	8 (5.1)	0	10 (2.3)
MC15	5 (2.1)	6 (3.9)	0	11 (2.6)
MC16	1 (0.4)	13 (8.3)	3 (8.8)	17 (4)
MC17	12 (5.1)	1 (0.6)	1 (2.9)	14 (3.3)
None MCs	9 (3.8)	6 (3.9)	2 (5.9)	17 (4)
Total	236	156	34	426

The predominant MC in each HIV status category is indicated in bold.

The distribution of environmental and clinical isolates among MCs was studied using two populations from Tokyo, Japan and Chiang Mai, Thailand. In both cases, the clinical and environmental isolates belonged to the same MCs (Figure 1D and Table 4). MC2 and MC8 were the common MCs in the Thai population. MC8 contained 26 out of 40 clinical isolates and 45 out of 58 environmental isolates, whereas MC2 contained 12 out of 40 clinical isolates and 8 out of 58 environmental isolates. Among the Japanese isolates, MC16 contained 16 out of 28 clinical isolates and 4 out of 9 environmental isolates, and MC2 contained 11 out of 28 clinical isolates and 3 out of 9 environmental isolates. The combined results of the Thai and Japanese populations showed a correlation between MCs and the clinical or environmental origin (*p* = 0.0004, Chi-square; *p* = 0.0001, Fisher exact test). However, no such correlation was observed within the Thai population from Chiang Mai (*p* = 0.0963, Chi-square; *p* = 0.0729, Fisher exact test) nor within the Japanese population from Tokyo (*p* = 0.9192, Chi-square; *p*>0.9999, Fisher exact test).

**Table 4:** Distribution of *C. neoformans* isolates from clinical and environmental samples from Thailand and Japan in microsatellite complexes (MCs).

MCs	Chiang Mai, Thailand		Tokyo, Japan		
	Clin. Isolates*n* (%)	Env. Isolates*n* (%)	Clin. Isolates*n* (%)	Env. Isolates*n* (%)	Total number of isolates*n* (%)
MC1	0	0	0	0	0
MC2	12 (30)	8 (13.8)	**11 (39.3)**	**3 (33.3)**	34 (25.2)
MC3	0	0	0	0	0
MC8	**26 (65)**	**45 (77.6)**	0	0	71 (52.6)
MC12	0	3 (5.2)	0	0	3 (2.2)
MC15	0	1 (1.7)	0	0	1 (0.7)
MC16	0	0	**16 (57.1)**	**4 (44.4)**	20 (14.8)
MC17	0	0	0	0	0
None MCs	2 (5)	1 (1.7)	1 (3.6)	2 (22.2)	6 (4.5)
Total	40	58	28	9	135

The predominant MCs in each sample type in these countries are indicated in bold.

### In vitro antifungal susceptibility testing

The MIC values of all *C. neoformans* var. *grubii* isolates for the seven antifungal compounds tested are listed in Table 5. Almost all cryptococcal isolates were susceptible to AMB, ITR, FLU, VOR, POS and ISA. Notably, 18 clinical isolates (3.7%) from Indonesia (*n* = 13), Thailand (*n* = 4) and China (*n* = 1) were resistant to 5FC with MIC ≥32 µg/ml (Table S3). Most of 5FC resistant isolates occurred in MC3 (*n* = 4), MC8 (*n* = 8) and MC17 (n = 5), and one belonged to MC2 (Table S4). Approximately 33.3% of all MC17 isolates were resistant to 5FC and this was found to be highly significant (*p*<0.0001, Chi-square; *p* = 0.0005, Fisher exact test) when compared to the presence of 3.9 and 9.5% of resistant isolates in MC8 and MC3, respectively. Ten FLU-resistant isolates (2%) occurred in different countries, including China (*n* = 2), India (*n* = 1), Indonesia (*n* = 5), and Thailand (*n* = 2) and belonged to MC2 (*n* = 3), MC3 (n = 2), MC8 (*n* = 2), MC17 (*n* = 2) and one isolate from India (number 25_17) that could not be assigned to any MC (*n* = 1) (Table S4). Five isolates from Indonesia (strain numbers 676, 1206, 1571, 3400, and 1048) that belonged to MC3 (*n* = 2), MC8 (*n* = 1) and MC17 (*n* = 2) were resistant to both 5FC and FLU. Isolates obtained from HIV-negative patients and HIV-positive patients did not differ in the susceptibility to three antifungal compounds, including 5FC (*p* = 0.0298, t-test), FLU (*p* = 0.0108, t-test) and VOR (*p*<0.0001, t-test). No significant differences were observed for ITR (*p* = 0.0002, t-test), POS (*p*<0.0001, t-test), and ISA (*p*<0.0001, t-test) when clinical and environmental isolates were compared. When all isolates were considered, the broadest ranges and highest MIC values were those of 5FC (<0.063 to >64 µg/ml), followed by FLU (0.125 to 32 µg/ml). The lowest MIC range was observed for ISA (<0.016 to 0.125 µg/ml), followed by POS, VOR and ITR (<0.016 to 0.5 µg/ml) (Table 5). Among the azoles, FLU showed the lowest activity (MIC_90_ = 4 µg/ml) and ISA the highest (MIC_90_ = 0.063 µg/ml) (Table 5).

**Table 5:** The MIC range, MIC_50_, MIC_90_, and geometric mean for all 493 *C. neoformans* isolates for seven antifungals according to clinical and environmental origin of isolates.

Isolates	Antifungal agent	MIC			
		Range	MIC_50_	Geometric Mean	MIC_90_
All *C. neoformans* isolates (*n* = 493)	Amphotericine B	0.063–1	0.25	0.251	0.5
	5-Flucytosine	<0.063–>64	4	3.483	8
	Fluconazole	0.125–32	2	2.294	4
	Itraconazole	<0.016–0.5	0.063	0.063	0.25
	Voriconazole	<0.016–0.5	0.063	0.049	0.125
	Posaconazole	<0.016–0.5	0.063	0.061	0.125
	Isavuconazole	<0.016–0.125	0.031	0.027	0.063
Isolates from HIV-positive patients (*n* = 236)	Amphotericine B	0.063–1	0.25	0.236	0.5
	5-Flucytosine	<0.063–>64	4	3.816	8
	Fluconazole	0.125–32	2	2.532	4
	Itraconazole	<0.016–0.5	0.063	0.062	0.25
	Voriconazole	<0.016–0.5	0.063	0.055	0.125
	Posaconazole	<0.016–0.5	0.063	0.062	0.125
	Isavuconazole	<0.016–0.125	0.031	0.025	0.063
Isolates from HIV-negative patients (*n* = 156)	Amphotericine B	0.063–1	0.25	0.253	0.5
	5-Flucytosine	0.25–32	4	3.091	8
	Fluconazole	0.125–16	2	2.072	4
	Itraconazole	<0.016–0.25	0.063	0.057	0.125
	Voriconazole	<0.016–0.25	0.063	0.036	0.125
	Posaconazole	<0.016–0.25	0.063	0.054	0.125
	Isavuconazole	<0.016–0.125	0.031	0.024	0.063
Clinical isolates from Thailand and Japan (*n* = 68)	Amphotericine B	0.063–0.5	0.25	0.291	0.5
	5-Flucytosine	0.5–8	4	3.433	8
	Fluconazole	0.25–32	2	2.452	4
	Itraconazole	<0.016–0.25	0.063	0.058	0.125
	Voriconazole	<0.016–0.5	0.063	0.070	0.125
	Posaconazole	<0.016–0.125	0.063	0.057	0.125
	Isavuconazole	<0.016–0.125	0.031	0.026	0.063
Environmental isolates from Thailand and Japan	Amphotericine B	0.125–0.5	0.25	0.286	0.5
(*n* = 67)	5-Flucytosine	0.063–8	4	2.933	4
	Fluconazole	0.125–8	2	2.106	4
	Itraconazole	<0.016–0.5	0.125	0.095	0.25
	Voriconazole	<0.016–0.125	0.063	0.055	0.125
	Posaconazole	<0.016–0.25	0.125	0.090	0.125
	Isavuconazole	<0.016–0.063	0.063	0.039	0.063

## Discussion

Microsatellites are an excellent tool to discriminate among *C. neoformans* var. *grubii* isolates [17], [33]. Indeed, 265 different genotypes were observed in this Asian collection of 493 isolates. Most isolates belonged to only eight microsatellite complexes (MCs), including four new ones. Simpson's index of diversity, however, showed that genetic diversity within each individual population and each MCs was very high.

The genotypic structure of the *C. neoformans* var. *grubii* isolates differed widely among the populations from the different countries. This finding supports the hypothesis that local geographic differences occur in Asia between *C. neoformans* var. *grubii* populations that could be the result from different founder effects and/or regional factors such as differences in the local environment and climate [34]–[36]. Recently, a population biology study using MLST analysis of *C. neoformans* var. *grubii* compared cryptococcal isolates from Thailand with a globally collected set of isolates. This study showed that the Asian population was genetically less diverse than those occurring in Africa and North America [37]. Furthermore, 15 of the 115 Chinese isolates (Table S1) were investigated previously by M13 PCR fingerprinting and identified as genotype VNIc [6]. All these isolates clustered in MC2, thus supporting the relatively limited genetic diversity among Chinese cryptococcal strains as seen by AFLP. Thus, it seems that the genetic diversity of cryptococcal isolates from Asian countries is lower than that of populations occurring in other parts of the world. The minimum spanning tree based on microsatellite analysis showed that MC8 contained mostly Thai isolates (Figure 1B), thus supporting the presence of limited genetic diversity among this population as has been described before [36]–[39]. However, other Thai isolates from HIV-infected patients and bird excreta from the North and Northeast occurred in MCs that comprised also isolates from China and Japan (i.e., MC2 and MC16). Can these findings be explained by assuming a relation with bird migration, especially the East Asia-Australian flyway, through which pathogenic microorganisms such as *C. neoformans* var. *grubii* may disperse between China and Thailand [40]? The scattered distribution of isolates from Kuwait and Qatar may be due to migration of foreign workers from Southeast Asia who may have carried isolates that were obtained from their country of origin, similar as has been demonstrated for African immigrants in France [41]. Further support for this hypothesis is that young children in USA acquired the organism from their surrounding environments [42] and we think that this also happens elsewhere.

Isolates belonging to MC8 contained mainly cryptococcal isolates from HIV-infected patients, whereas MC2 and MC16 comprised mostly isolates from non-HIV-infected patients (Figure 1C). A recent report from Vietnam revealed two genotypic clusters based on AFLP analysis, viz., VNIγ and VNIδ, that contained isolates from HIV-negative and positive patients, respectively [10]. Thus genotypic differences were seen between both patient categories based on AFLP analysis and this observation is reminiscent to the differences that we observed in the current microsatellite data. However, no comparison could be made between the AFLP- and microsatellite typing because, unfortunately, none of the Vietnamese isolates was available for our studies.

In Japan and Thailand, environmental isolates co-occurred with clinical isolates in MC2, MC8, and MC16 (Figure 1A, C and D, and Table 3), and these results suggest a genetic relatedness between environmental and clinical cryptococcal isolates in these countries. Such a relationship has been suggested before [34], [43]–[46], but is at stake with a recent analysis from Cuba where the clinical isolates largely belonged to a different MC than those obtained from the environment [17]. At present, the observed differences between the genetic relationship of environmental and clinical isolates in different parts of the globe are not easily explained, but it may be that different environmental niches are occupied in various locales.

Susceptibility analysis of 493 *C. neoformans* isolates to seven antifungals yielded no change in MIC ranges and MIC_50_ and MIC_90_ values of AMB and FLU when compared to previous studies [47]–[49]. The MIC ranges and values for ITR and the three novel antifungal agents POS, ISA, and VOR were slightly changed to those reported previously [22], [48]–[49]. The MIC_50_ and MIC_90_ values for ISA were lower than those observed for the other antifungal agents, thus corroborating previous observations [48], [49]

Importantly, resistance in approximately 4% of the isolates to 5FC was observed. Most 5FC-resistant isolates came from Indonesia and Thailand where this drug is not in use [50]–[51]. In our study, we found that approximately 35% of all MC17 isolates were resistant to 5FC. These findings suggest that intrinsic resistance to 5FC occurs among Southeast Asian isolates of *C. neoformans*, especially in MC17 and in Indonesia. Intrinsic resistance to 5FC in *C. neoformans* is uncommon and occurs with a low incidence of approximately 1–2% as reported from the USA in the 1990s [52]–[53]. The reason for this relative high number of 5FC resistant isolates in the Southeast Asian region is not clear, and needs further study. Furthermore, resistance to FLU was observed. The FLU-resistant isolates occurred in different countries, including China, India, Indonesia, and Thailand. *C. neoformans* may develop resistance to FLU after treatment [52], [54], but the molecular origin of the resistance mechanism among the Asian strains remains unknown.

Five out of 10 FLU resistant isolates (2 from MC3, 1 from MC8, and 2 from MC17) from Indonesia were also not susceptible to 5FC. Our findings suggested that isolates in certain MCs, especially MC17, may be prone to a decreased susceptibility to antifungals, especially FLU and 5FC. The observed double resistance to 5FC and FLU has not been reported before, and may pose a risk for the patients infected with such isolates. Isolates from HIV-infected and non-HIV-infected patients did not differ in MIC values of 5FC, FLU, and VOR, but the MIC ranges of isolates from HIV-infected patients were broader than those of isolates from non-HIV-infected patients. The highest MIC values of isolates from HIV-infected patients were 16 times higher for 5FC and FLU and two to four times as high as those from non-HIV-infected patients, respectively.

This is the first extensive report on *in vitro* antifungal susceptibility and genotyping of clinical and environmental isolates of *C. neoformans* from several Asian countries. Based on the *in vitro* susceptibility test results, the patients in the region seem to receive appropriate antifungal therapy with AMB plus 5FC for induction therapy followed by consolidation/maintenance therapy of FLU. AMB is still the most effective agent to treat an infection with *C. neoformans* [4]. A Chinese study showed that the risk of death in patients with cryptococcosis who did not receive AMB-based initial therapy was about 7–9 times higher than those given AMB [54]. Another study from Thailand used different combinations of antifungal therapies, i.e., AMB alone, AMB plus 5FC, AMB plus FLU, or triple antifungals (AMB plus 5FC and FLU) to treat cryptococcal meningitis showed that treatment of AMB combined with 5FC remains a powerful treatment strategy that results in rapid clearance of the pathogen [55]. Our study did not reveal a significant change in the MICs of AMB for *C. neoformans*, and therefore, initial treatment with AMB remains the recommended choice. However, given the well-tolerated nature and a excellent activity against *Cryptococcus* strains, the new generation of triazoles may become an important addition to the currently used antifungals.

In summary, genotypic differences in microsatellite patterns occur between *C. neoformans* populations from the Asian countries studied. Most of the countries had a unique distribution of MCs but the overall genetic diversity estimated by Simpson's index of diversity was high. Good *in vitro* antifungal activity was observed, but 5FC and FLU resistant, as well as 5FC/FLU double resistant isolates occurred.

## Supporting Information

Table S1 **Origin of** ***Cryptococcus neoformans*** **var.** ***grubii*** **isolates and clinical background information of the patients.**
(DOC)

Table S2 **Environmental** ***Cryptococcus neoformans*** **var.** ***grubii*** **isolates from Thailand and Japan.**
(DOC)

Table S3 **The MIC range, MIC_50_, MIC_90_, and geometric mean for clinical isolates of** ***C. neoformans*** **in each country.**
(DOC)

Table S4 **The MIC range, MIC_50_, MIC_90_, and geometric mean for the different microsatellite complexes (MCs) of** ***C. neoformans***.
(DOC)
