# Supplementary material for: Resistance of Asian Cryptococcus neoformans Serotype A Is Confined to Few Microsatellite Genotypes
Source: PLoS One. 2012 Mar 13;7(3):e32868. doi: 10.1371/journal.pone.0032868 (PMC3302784; doi:10.1371/journal.pone.0032868)
Supplement: Table S1 — Origin of Cryptococcus neoformans var. grubii isolates and clinical background information of the patients. (DOC) [file pone.0032868.s001.doc]

**Table S1. Origin of *Cryptococcus* *neoformans* var. *grubii*** isolates and clinical background information of the patients

| Isolate | Mating/  Serotype | Source | Geographic origin | HIV status | MCs |
| --- | --- | --- | --- | --- | --- |
| CN5019 | αA | Blood | Chiang Rai, Thailand | Positive | MC8 |
| CN5018 | αA | Blood | Chiang Rai, Thailand | Positive | MC2 |
| CN5017 | αA | CSF | Chiang Rai, Thailand | Positive | MC8 |
| CN5015 | αA | CSF | Chiang Rai, Thailand | Positive | MC2 |
| CN5014 | αA | Blood | Chiang Rai, Thailand | Positive | MC8 |
| CN5013 | αA | CSF | Chiang Rai, Thailand | Positive | MC8 |
| CN5012 | αA | CSF | Chiang Rai, Thailand | Positive | MC2 |
| CN5011 | αA | Blood | Chiang Rai, Thailand | Positive | MC8 |
| CN5010 | αA | Blood | Chiang Rai, Thailand | Positive | MC8 |
| CN5009 | αA | Blood | Chiang Rai, Thailand | Positive | MC8 |
| CN5008 | αA | CSF | Chiang Rai, Thailand | Positive | MC16 |
| CN5007 | αA | CSF | Chiang Rai, Thailand | Positive | MC17 |
| CN5005 | αA | Blood | Chiang Rai, Thailand | Positive | MC8 |
| CN5003 | αA | Blood | Chiang Rai, Thailand | Positive | MC8 |
| CN5002 | αA | Blood | Chiang Rai, Thailand | Positive | MC8 |
| CN5001 | αA | CSF | Chiang Rai, Thailand | Positive | MC8 |
| CN4998 | αA | CSF | Chiang Mai, Thailand | Positive | MC8 |
| CN4995 | αA | CSF | Chiang Mai, Thailand | Positive | MC8 |
| CN4993 | αA | CSF | Chiang Mai, Thailand | Positive | MC2 |
| CN4989 | αA | CSF | Chiang Mai, Thailand | Positive | MC8 |
| CN4988 | αA | CSF | Chiang Mai, Thailand | Positive | MC8 |
| CN4987 | αA | CSF | Chiang Mai, Thailand | Positive | MC8 |
| CN4983 | αA | CSF | Chiang Mai, Thailand | Positive | MC2 |
| CN4980 | αA | CSF | Chiang Mai, Thailand | Positive | MC2 |
| CN4977 | αA | CSF | Chiang Mai, Thailand | Positive | MC2 |
| CN4970 | αA | CSF | Chiang Mai, Thailand | Positive | MC8 |
| CN4968 | αA | CSF | Chiang Mai, Thailand | Positive | MC8 |
| CN4967 | αA | CSF | Chiang Mai, Thailand | Positive | MC2 |
| CN4964 | αA | CSF | Chiang Mai, Thailand | Positive | MC8 |
| CN4960 | αA | CSF | Chiang Rai, Thailand | Positive | MC2 |
| CN4957 | αA | CSF | Chiang Rai, Thailand | Positive | MC8 |
| CN4956 | αA | CSF | Chiang Rai, Thailand | Positive | MC8 |
| CN4955 | αA | BAL | The North,Thailand | Positive | MC8 |
| CN4954 | αA | CSF | Lampang, Thailand | Positive | MC8 |
| CN4952 | αA | CSF | Tak, Thailand | Positive | MC8 |
| CN4950 | αA | CSF | Lampoon, Thailand | Positive | MC8 |
| CN4949 | αA | CSF | Lampoon, Thailand | Positive | MC8 |
| CN4948 | αA | CSF | Chiang Mai, Thailand | Positive | MC2 |
| CN4947 | αA | CSF | Chiang Rai, Thailand | Positive | MC8 |
| CN4946 | αA | CSF | Chiang Mai, Thailand | Positive | MC2 |
| CN4945 | αA | CSF | Chiang Rai, Thailand | Positive | MC8 |
| CN4944 | αA | CSF | Chiang Mai, Thailand | Positive | MC8 |
| CN4943 | αA | CSF | Chiang Rai, Thailand | Positive | MC8 |
| CN4942 | αA | CSF | Lampang, Thailand | Positive | MC8 |
| CN4941 | αA | CSF | The North,Thailand | Positive | MC8 |
| CN4940 | αA | CSF | The North,Thailand | Positive | MC8 |
| CN4938 | αA | CSF | Chiang Mai, Thailand | Positive | MC8 |
| CN4937 | αA | CSF | Chiang Mai, Thailand | Positive | MC8 |
| CN4936 | αA | CSF | Chiang Mai, Thailand | Positive | MC8 |
| CN4934 | αA | CSF | Chiang Mai, Thailand | Positive | MC8 |
| CN4933 | αA | CSF | Chiang Mai, Thailand | Positive | MC8 |
| CN4932 | αA | CSF | Chiang Mai, Thailand | Positive | MC8 |
| CN4931 | αA | CSF | Chiang Mai, Thailand | Positive | MC8 |
| CN4927 | αA | CSF | Chiang Mai, Thailand | Positive | MC8 |
| CN4926 | αA | CSF | Chiang Rai, Thailand | Positive | MC8 |
| CN4924 | αA | CSF | Chiang Mai, Thailand | Positive | MC2 |
| CN4921 | αA | CSF | Mae Hong Son, Thailand | Positive | MC2 |
| CN4920 | αA | CSF | Chiang Mai, Thailand | Positive | MC2 |
| CN4919 | αA | CSF | Chiang Rai, Thailand | Positive | MC8 |
| CN4918 | αA | CSF | Chiang Rai, Thailand | Positive | MC8 |
| CN4917 | αA | CSF | Chiang Rai, Thailand | Positive | MC8 |
| CN4916 | αA | CSF | Chiang Mai, Thailand | Positive | MC2 |
| CN4915 | αA | CSF | Chiang Mai, Thailand | Positive | MC8 |
| CN4914 | αA | CSF | Chiang Mai, Thailand | Positive | MC8 |
| CN4909 | αA | CSF | Chiang Mai, Thailand | Positive | MC8 |
| CN4907 | αA | CSF | Chiang Mai, Thailand | Positive | MC8 |
| CN4906 | αA | CSF | Chiang Mai, Thailand | Positive | MC2 |
| CN4905 | αA | CSF | Chiang Mai, Thailand | Positive | MC8 |
| CN4904 | αA | CSF | Chiang Mai, Thailand | Positive | MC8 |
| CN4903 | αA | CSF | Chiang Rai, Thailand | Positive | MC8 |
| CN4902 | αA | CSF | Chiang Mai, Thailand | Positive | MC8 |
| CN4901 | αA | CSF | Chiang Mai, Thailand | Positive | MC8 |
| CN49008 | αA | CSF | Chiang Mai, Thailand | Positive | MC8 |
| CN49006 | αA | CSF | Chiang Mai, Thailand | Positive | None |
| CN49005 | αA | CSF | Chiang Mai, Thailand | Positive | None |
| CN49004 | αA | CSF | Chiang Mai, Thailand | Positive | MC2 |
| 4-319 | αA | Unknown | Khon Kaen, Thailand | Unknown | MC8 |
| 4-187 | αA | Unknown | Khon Kaen, Thailand | Unknown | MC8 |
| CN48 | αA | Unknown | Khon Kaen, Thailand | Unknown | MC2 |
| 50NC2 | αA | CSF | Nan, Thailand | Positive | MC8 |
| 269 | αA | Unknown | Khon Kaen, Thailand | Unknown | MC8 |
| 50NC5 | αA | CSF | Nan, Thailand | Positive | MC8 |
| 4-315 | αA | Unknown | Khon Kaen, Thailand | Unknown | MC8 |
| 1-587 | αA | Unknown | Khon Kaen, Thailand | Unknown | MC8 |
| 11112 | αA | Unknown | Khon Kaen, Thailand | Unknown | MC8 |
| 4_9 | αA | Unknown | Khon Kaen, Thailand | Unknown | MC8 |
| 11109 | αA | Unknown | Khon Kaen, Thailand | Unknown | MC8 |
| 4-231 | αA | Unknown | Khon Kaen, Thailand | Unknown | MC8 |
| 1219 | αA | Unknown | Khon Kaen, Thailand | Unknown | MC8 |
| P6 | αA | Unknown | Khon Kaen, Thailand | Unknown | MC8 |
| 4_83 | αA | Unknown | Khon Kaen, Thailand | Unknown | MC8 |
| 1-588 | αA | Unknown | Khon Kaen, Thailand | Unknown | MC8 |
| 1-488 | αA | Unknown | Khon Kaen, Thailand | Unknown | MC2 |
| 4-253 | αA | Unknown | Khon Kaen, Thailand | Unknown | MC8 |
| 4-202 | αA | Unknown | Khon Kaen, Thailand | Unknown | MC8 |
| 1-846 | αA | Unknown | Khon Kaen, Thailand | Unknown | MC8 |
| 50NC1 | αA | CSF | Nan, Thailand | Positive | MC8 |
| 4-381 | αA | Unknown | Khon Kaen, Thailand | Unknown | MC8 |
| 1-489 | αA | Unknown | Khon Kaen, Thailand | Unknown | MC8 |
| 20662-07 | αA | Blood | Songkhla, Thailand | Positive | MC8 |
| 28170-07 | αA | CSF | Songkhla, Thailand | Positive | MC8 |
| 2551-07 | αA | Unknown | Songkhla, Thailand | Positive | MC8 |
| 2550 II-07 | αA | Blood | Songkhla, Thailand | Positive | MC8 |
| 2461-07 | αA | CSF | Songkhla, Thailand | Positive | MC8 |
| 1111I-08 | αA | Blood | Patani, Thailand | Negative | MC8 |
| 1291-09 | αA | Blood | Patani, Thailand | Negative | MC8 |
| 2895I-08 | αA | Blood | Patani, Thailand | Negative | MC8 |
| 4500-07 | αA | Blood | Patani, Thailand | Positive | MC8 |
| CM1 | αA | CSF | Ubon Ratchatani, Thailand | Positive | MC8 |
| CM2 | αA | CSF | Ubon Ratchatani, Thailand | Positive | MC8 |
| CM3 | αA | CSF | Ubon Ratchatani, Thailand | Positive | MC8 |
| CM4 | αA | CSF | Ubon Ratchatani, Thailand | Positive | MC8 |
| CM5 | αA | CSF | Ubon Ratchatani, Thailand | Positive | MC8 |
| CM6 | αA | CSF | Ubon Ratchatani, Thailand | Positive | MC8 |
| CM7 | αA | CSF | Ubon Ratchatani, Thailand | Positive | None |
| CM8 | αA | CSF | Ubon Ratchatani, Thailand | Positive | MC8 |
| CM9 | αA | CSF | Ubon Ratchatani, Thailand | Positive | MC8 |
| CM10 | αA | CSF | Ubon Ratchatani, Thailand | Positive | MC8 |
| CM11 | αA | CSF | Ubon Ratchatani, Thailand | Positive | MC8 |
| CM12 | αA | CSF | Ubon Ratchatani, Thailand | Positive | MC8 |
| CM13 | αA | CSF | Ubon Ratchatani, Thailand | Positive | MC8 |
| CM14 | αA | CSF | Ubon Ratchatani, Thailand | Positive | MC8 |
| CM15 | αA | CSF | Ubon Ratchatani, Thailand | Positive | MC8 |
| CM16 | αA | CSF | Ubon Ratchatani, Thailand | Positive | MC2 |
| CM17 | αA | CSF | Ubon Ratchatani, Thailand | Positive | MC8 |
| CM18 | αA | CSF | Ubon Ratchatani, Thailand | Positive | MC8 |
| CM20 | αA | CSF | Ubon Ratchatani, Thailand | Positive | MC8 |
| CM21 | αA | CSF | Ubon Ratchatani, Thailand | Positive | MC17 |
| CM22 | αA | CSF | Ubon Ratchatani, Thailand | Positive | MC8 |
| CM23 | αA | CSF | Ubon Ratchatani, Thailand | Positive | MC8 |
| CM24 | αA | CSF | Ubon Ratchatani, Thailand | Positive | MC8 |
| CM25 | αA | CSF | Ubon Ratchatani, Thailand | Positive | MC8 |
| CM26 | αA | CSF | Ubon Ratchatani, Thailand | Positive | MC8 |
| CM27 | αA | CSF | Ubon Ratchatani, Thailand | Positive | MC8 |
| CM28 | αA | CSF | Ubon Ratchatani, Thailand | Positive | MC8 |
| CM29 | αA | CSF | Ubon Ratchatani, Thailand | Positive | MC8 |
| CM30 | αA | CSF | Ubon Ratchatani, Thailand | Positive | MC8 |
| CM32 | αA | CSF | Ubon Ratchatani, Thailand | Positive | MC8 |
| CM33 | αA | CSF | Ubon Ratchatani, Thailand | Positive | MC8 |
| CM34 | αA | CSF | Ubon Ratchatani, Thailand | Positive | MC8 |
| CM35 | αA | CSF | Ubon Ratchatani, Thailand | Positive | MC8 |
| CM36 | αA | CSF | Ubon Ratchatani, Thailand | Positive | MC8 |
| CM37 | αA | CSF | Ubon Ratchatani, Thailand | Positive | MC8 |
| CM38 | αA | CSF | Ubon Ratchatani, Thailand | Positive | MC8 |
| CM39 | αA | CSF | Ubon Ratchatani, Thailand | Positive | MC8 |
| CM40 | αA | CSF | Ubon Ratchatani, Thailand | Positive | MC8 |
| CM41 | αA | CSF | Ubon Ratchatani, Thailand | Positive | MC8 |
| CM42 | αA | CSF | Ubon Ratchatani, Thailand | Positive | MC8 |
| CM43 | αA | CSF | Ubon Ratchatani, Thailand | Positive | MC8 |
| CM44 | αA | CSF | Ubon Ratchatani, Thailand | Positive | MC8 |
| CM45 | αA | CSF | Ubon Ratchatani, Thailand | Positive | MC8 |
| CM46 | αA | CSF | Ubon Ratchatani, Thailand | Positive | MC8 |
| CM47 | αA | CSF | Ubon Ratchatani, Thailand | Positive | MC8 |
| CM48 | αA | CSF | Ubon Ratchatani, Thailand | Positive | MC8 |
| CM49 | αA | CSF | Ubon Ratchatani, Thailand | Positive | MC8 |
| CM50 | αA | CSF | Ubon Ratchatani, Thailand | Positive | MC2 |
| CM51 | αA | CSF | Ubon Ratchatani, Thailand | Positive | MC8 |
| CM52 | αA | CSF | Ubon Ratchatani, Thailand | Positive | MC2 |
| CM55 | αA | CSF | Ubon Ratchatani, Thailand | Positive | MC8 |
| CM56 | αA | CSF | Ubon Ratchatani, Thailand | Positive | MC8 |
| CM57 | αA | CSF | Ubon Ratchatani, Thailand | Positive | MC8 |
| CM58 | αA | CSF | Ubon Ratchatani, Thailand | Positive | MC8 |
| CM59 | αA | CSF | Ubon Ratchatani, Thailand | Positive | MC8 |
| CM60 | αA | CSF | Ubon Ratchatani, Thailand | Positive | MC8 |
| CM61 | αA | CSF | Ubon Ratchatani, Thailand | Positive | MC17 |
| CM63 | αA | CSF | Ubon Ratchatani, Thailand | Positive | MC8 |
| CM64 | αA | CSF | Ubon Ratchatani, Thailand | Positive | MC8 |
| 9104 | αA | Skin/Sputum | Tokyo, Japan | Negative | MC2 |
| 9106 | αA | CSF | Tokyo, Japan | Negative | MC16 |
| 9107 | αA | Unknown | Tokyo, Japan | Negative | MC2 |
| 9108 | αA | Skin | Tokyo, Japan | Negative | MC2 |
| 9111 | αA | CSF | Tokyo, Japan | Negative | MC2 |
| 9114 | αA | Unknown | Tokyo, Japan | Negative | MC16 |
| 9165 | αA | CSF | Tokyo, Japan | Negative | MC2 |
| 9166 | αA | Skin | Tokyo, Japan | Negative | MC16 |
| 9167 | αA | CSF/Skin | Tokyo, Japan | Negative | MC2 |
| 9170 | αA | Skin | Tokyo, Japan | Negative | MC16 |
| 9172 | αA | CSF | Tokyo, Japan | Unknown | MC2 |
| 9173 | αA | CSF | Tokyo, Japan | Negative | MC16 |
| 9174 | αA | CSF | Tokyo, Japan | Negative | MC16 |
| 9179 | αA | Unknown | Tokyo, Japan | Negative | MC16 |
| 9197 | αA | Unknown | Tokyo, Japan | Unknown | MC16 |
| 9198 | αA | Unknown | Tokyo, Japan | Unknown | MC2 |
| 9199 | αA | Unknown | Tokyo, Japan | Unknown | None |
| 9204 | αA | Unknown | Tokyo, Japan | Unknown | MC2 |
| 9205 | αA | Unknown | Tokyo, Japan | Unknown | MC2 |
| 9213 | αA | Unknown | Tokyo, Japan | Unknown | MC16 |
| 9217 | αA | Unknown | Tokyo, Japan | Unknown | MC16 |
| 9237 | αA | Skin | Tokyo, Japan | Negative | MC16 |
| 9238 | αA | CSF | Tokyo, Japan | Negative | MC16 |
| 9239 | αA | Skin | Tokyo, Japan | Negative | MC16 |
| 9251 | αA | Skin | Tokyo, Japan | Negative | MC16 |
| 9263 | αA | Skin | Tokyo, Japan | Negative | MC16 |
| 9264 | αA | Skin | Tokyo, Japan | Negative | MC16 |
| 9265 | αA | Skin | Tokyo, Japan | Negative | MC2 |
| 132 | αA | CSF | Jakarta, Indonesia | Positive | MC3 |
| 267 | αA | CSF | Jakarta, Indonesia | Positive | MC8 |
| 268 | αA | CSF | Jakarta, Indonesia | Positive | MC3 |
| 328 | αA | CSF | Jakarta, Indonesia | Positive | MC3 |
| 544 | αA | CSF | Jakarta, Indonesia | Positive | MC17 |
| 597 | αA | CSF | Jakarta, Indonesia | Positive | MC8 |
| 612 | αA | CSF | Jakarta, Indonesia | Positive | MC3 |
| 676 | αA | CSF | Jakarta, Indonesia | Positive | MC3 |
| 778 | αA | CSF | Jakarta, Indonesia | Positive | MC8 |
| 1019 | αA | CSF | Jakarta, Indonesia | Positive | MC8 |
| 1051 | αA | CSF | Jakarta, Indonesia | Positive | MC15 |
| 1116 | αA | CSF | Jakarta, Indonesia | Positive | MC3 |
| 1200 | αA | CSF | Jakarta, Indonesia | Positive | MC17 |
| 1206 | αA | CSF | Jakarta, Indonesia | Positive | MC17 |
| 1336 | αA | CSF | Jakarta, Indonesia | Positive | MC17 |
| 1462 | αA | CSF | Jakarta, Indonesia | Positive | MC8 |
| 1571 | αA | CSF | Jakarta, Indonesia | Positive | MC3 |
| 2126 | αA | CSF | Jakarta, Indonesia | Positive | MC17 |
| 2339 | αA | CSF | Jakarta, Indonesia | Positive | MC8 |
| 2478 | αA | CSF | Jakarta, Indonesia | Positive | MC8 |
| 2594 | αA | CSF | Jakarta, Indonesia | Positive | None |
| 2606 | αA | CSF | Jakarta, Indonesia | Positive | MC8 |
| 3187 | αA | CSF | Jakarta, Indonesia | Positive | MC17 |
| 3281 | αA | Unknown | Jakarta, Indonesia | Unknown | MC8 |
| 3400 | αA | CSF | Jakarta, Indonesia | Positive | MC8 |
| 3594 | αA | CSF | Jakarta, Indonesia | Positive | MC8 |
| 3634 | αA | CSF | Jakarta, Indonesia | Positive | MC8 |
| 1048 | αA | Unknown | Jakarta, Indonesia | Unknown | MC17 |
| Jakarta 1051 | αA | CSF | Jakarta, Indonesia | Positive | MC3 |
| 2597 | αA | CSF | Jakarta, Indonesia | Positive | MC8 |
| 264 | αA | CSF | Jakarta, Indonesia | Positive | None |
| Jakarta (P) | αA | Blood | Jakarta, Indonesia | Positive | MC3 |
| Jakarta(H) | αA | Blood | Jakarta, Indonesia | Positive | MC8 |
| Jakarta (KT) | αA | Skin | Jakarta, Indonesia | Positive | MC8 |
| Jakarta KLT | αA | Skin | Jakarta, Indonesia | Positive | MC8 |
| Jakarta RTL | αA | Skin | Jakarta, Indonesia | Positive | MC8 |
| Jakarta | αA | CSF | Jakarta, Indonesia | Positive | MC17 |
| CrOE | αA | CSF | Jakarta, Indonesia | Negative | MC17 |
| Cr755 | αA | CSF | Jakarta, Indonesia | Positive | MC17 |
| Cr2231 | αA | CSF | Jakarta, Indonesia | Positive | MC17 |
| 25_228 | αA | CSF | Chandigarh, India | Positive | MC3 |
| 25_229 | αA | CSF | Chandigarh, India | Positive | MC15 |
| 25_237 | αA | CSF | Chandigarh, India | Positive | MC3 |
| 25_240 | αA | CSF | Chandigarh, India | Negative | None |
| 25_244 | αA | CSF | Chandigarh, India | Positive | MC1 |
| 25_261 | αA | CSF | Chandigarh, India | Positive | MC3 |
| 25_266 | αA | CSF | Chandigarh, India | Negative | MC15 |
| 25_272 | αA | BAL | Chandigarh, India | Positive | MC3 |
| 25_277 | αA | CSF | Chandigarh, India | Positive | MC3 |
| 25_290 | αA | CSF | Chandigarh, India | Positive | MC3 |
| 25_291 | αA | CSF | Chandigarh, India | Negative | MC2 |
| 25_292 | αA | CSF | Chandigarh, India | Positive | MC3 |
| 25_296 | αA | CSF | Chandigarh, India | Positive | None |
| 25_298 | αA | CSF | Chandigarh, India | Negative | MC15 |
| 25_299 | αA | CSF | Chandigarh, India | Negative | MC3 |
| 25_302 | αA | CSF | Chandigarh, India | Positive | MC3 |
| 25_304 | αA | CSF | Chandigarh, India | Positive | MC3 |
| 25_308 | αA | CSF | Chandigarh, India | Positive | MC3 |
| 25_311 | αA | CSF | Chandigarh, India | Negative | None |
| 25_312 | αA | CSF | Chandigarh, India | Positive | MC3 |
| 25_313 | αA | CSF | Chandigarh, India | Positive | MC3 |
| 25_316 | αA | CSF | Chandigarh, India | Negative | MC8 |
| 25_328 | αA | CSF | Chandigarh, India | Positive | None |
| 25_336 | αA | CSF | Chandigarh, India | Positive | MC3 |
| 25_337 | αA | CSF | Chandigarh, India | Positive | MC3 |
| 25_334 | αA | CSF | Chandigarh, India | Positive | MC3 |
| 25_339 | αA | CSF | Chandigarh, India | Negative | MC3 |
| 25_340 | αA | CSF | Chandigarh, India | Negative | MC15 |
| 25_344 | αA | BAL | Delhi, India | Positive | MC3 |
| 25_356 | αA | CSF | Chandigarh, India | Positive | MC3 |
| 25_357 | αA | CSF | Chandigarh, India | Positive | MC15 |
| 25_358 | αA | CSF | Chandigarh, India | Positive | MC3 |
| 25_239 | αA | CSF | Chandigarh, India | Positive | MC3 |
| 25_104 | αA | CSF | Chandigarh, India | Positive | MC1 |
| 25_105 | αA | CSF | Chandigarh, India | Negative | MC1 |
| 25_110 | αA | CSF | Chandigarh, India | Negative | MC1 |
| 25_14 | αA | Unknown | India | Unknown | MC1 |
| 25_17 | αA | Blood | Chandigarh, India | Negative | None |
| 25_18 | αA | CSF | Chandigarh, India | Negative | MC12 |
| 25_33 | αA | CSF | Chandigarh, India | Negative | MC3 |
| 25_341 | αA | CSF | Chandigarh, India | Positive | MC3 |
| 25_355 | αA | CSF | Chandigarh, India | Negative | MC12 |
| 25_365 | αA | CSF | Chandigarh, India | Positive | MC1 |
| 25_367 | αA | CSF | Chandigarh, India | Positive | MC3 |
| 25_368 | αA | CSF | Chandigarh, India | Positive | MC3 |
| 25_369 | αA | CSF | Assam, India | Positive | MC1 |
| 25_370 | αA | CSF | Assam, India | Positive | MC15 |
| 25_371 | αA | CSF | Assam, India | Negative | MC3 |
| 25_372 | αA | CSF | Assam, India | Positive | MC8 |
| 25_40 | αA | Unknown | India | Unknown | MC1 |
| 25_49 | αA | CSF | Chandigarh, India | Negative | MC3 |
| 25_50 | αA | CSF | Chandigarh, India | Negative | MC3 |
| 25_52 | αA | CSF | Chandigarh, India | Negative | MC1 |
| 25_53 | αA | CSF | Karnataka, India | Negative | MC1 |
| 25_61 | αA | CSF | Calcutta, India | Positive | MC1 |
| 25_62 | αA | CSF | Karnataka, India | Negative | MC1 |
| 25_63 | αA | CSF | Karnataka, India | Positive | MC1 |
| 25_78 | αA | Unknown | India | Unknown | MC3 |
| 25_84 | αA | Blood | Chandigarh, India | Negative | MC1 |
| 25_86 | αA | CSF | Karnataka, India | Negative | MC3 |
| 25_373 | αA | CSF | Chandigarh, India | Positive | MC8 |
| 1185/04 | αA | Endotracheal secretion | Kuwait City, Kuwait | Negative | MC2 |
| 110/99 | αA | Blood | Kuwait City, Kuwait | Negative | None |
| 2365/08 | αA | CSF | Kuwait City, Kuwait | Negative | None |
| 481/03 | αA | CSF | Kuwait City, Kuwait | Positive | MC3 |
| 194/96 | αA | Lymph node biopsy | Kuwait City, Kuwait | Positive | MC8 |
| 1589/04 | αA | CSF | Kuwait City, Kuwait | Negative | MC15 |
| 177/02 | αA | Wound swab | Kuwait City, Kuwait | Negative | MC15 |
| 201/95 | αA | Lumbar swelling aspirate | Kuwait City, Kuwait | Negative | MC2 |
| 8/92 | αA | CSF | Kuwait City, Kuwait | Unknown | None |
| 200/16 | αA | Lymph node biopsy | Kuwait City, Kuwait | Positive | MC8 |
| 1608000352 | αA | CSF | Doha, Qatar | Negative | MC15 |
| 1609290340 | αA | CSF | Doha, Qatar | Negative | MC2 |
| 1608000894 | αA | CSF | Doha, Qatar | Positive | MC8 |
| 1607001262 | αA | CSF | Doha, Qatar | Positive | MC2 |
| 1605202443 | αA | CSF | Doha, Qatar | Positive | MC15 |
| WH001 | αA | CSF | Shanghai, China | Negative | MC3 |
| WH003 | αA | Sputum | Fujian, China | Negative | MC2 |
| WH004 | αA | CSF | Anhui, China | Negative | MC2 |
| WH005 | αA | CSF | Jiangsu, China | Negative | MC2 |
| WH006 | αA | CSF | Henan, China | Negative | MC2 |
| WH007 | αA | CSF | Beijing, China | Negative | MC2 |
| WH008 | αA | CSF | Hainan, China | Negative | MC2 |
| WH009 | αA | CSF | Hunan, China | Negative | MC2 |
| WH010 | αA | CSF | Jiangsu, China | Negative | MC2 |
| WH011 | αA | CSF | Anhui, China | Negative | MC2 |
| WH012 | αA | CSF | Shanghai, China | Negative | MC2 |
| WH013 | αA | CSF | Zhejang, China | Negative | MC2 |
| WH014 | αA | CSF | Shandong, China | Negative | MC2 |
| WH015 | αA | Sputum | Guangdong, China | Negative | MC2 |
| WH016 | αA | CSF | Guangdong, China | Negative | MC2 |
| WH017 | αA | CSF | Guangdong, China | Negative | MC2 |
| WH018 | αA | CSF | Guangdong, China | Negative | MC2 |
| WH019 | αA | CSF | Guangdong, China | Negative | MC2 |
| WH020 | αA | Blood | Guangdong, China | Negative | MC2 |
| WH021 | αA | Blood | Guangdong, China | Negative | MC2 |
| WH022 | αA | CSF | Guangdong, China | Negative | MC2 |
| WH023 | αA | CSF | Guangdong, China | Negative | MC2 |
| WH025 | αA | CSF | Shanghai, China | Negative | MC2 |
| WH026 | αA | CSF | Guangdong, China | Negative | MC2 |
| WH027 | αA | CSF | Shanghai, China | Negative | MC2 |
| WH028 | αA | CSF | Guangdong, China | Negative | MC2 |
| WH029 | αA | CSF | Jiangsu, China | Negative | MC12 |
| WH030 | αA | CSF | Jiangsu, China | Negative | MC2 |
| WH031 | αA | Lung | Shanghai, China | Negative | MC2 |
| WH032 | αA | CSF | Zhejang, China | Negative | MC2 |
| WH033 | αA | Skin | Shanghai, China | Negative | MC2 |
| WH034 | αA | CSF | Jiangsu, China | Negative | MC2 |
| WH035 | αA | CSF | Henan, China | Negative | MC2 |
| WH036 | αA | CSF | Shanghai, China | Negative | MC2 |
| WH037 | αA | CSF | Henan, China | Negative | MC12 |
| WH040 | αA | CSF | Shanghai, China | Negative | MC2 |
| WH041 | αA | CSF | Guangdong, China | Negative | MC2 |
| WH042 | αA | CSF | Shanghai, China | Negative | MC2 |
| WH043 | αA | Sputum | Shanghai, China | Negative | MC2 |
| WH044 | αA | CSF | Beijing, China | Negative | MC2 |
| WH046 | αAαD | CSF | Shandong, China | Negative | MC12 |
| WH047 | αA | CSF | Guangdong, China | Negative | MC2 |
| WH048 | αA | CSF | Shanghai, China | Negative | MC2 |
| WH049 | αA | CSF | Shanghai, China | Positive | MC2 |
| WH050 | αA | CSF | Jiangsu, China | Negative | MC2 |
| WH051 | αA | CSF | Guangdong, China | Negative | MC2 |
| WH052 | αA | CSF | SanXi, China | Negative | MC2 |
| WH054 | αA | CSF | Guangdong, China | Negative | MC2 |
| WH055 | αA | CSF | Hubei, China | Negative | MC2 |
| WH057 | αA | CSF | Shanghai, China | Negative | MC2 |
| WH058 | αA | Skin | Guangdong, China | Negative | MC2 |
| WH059 | αA | Blood | Guangdong, China | Negative | MC2 |
| WH060 | αA | CSF | Guangdong, China | Negative | MC2 |
| WH061 | αA | CSF | Guangdong, China | Negative | MC2 |
| WH062 | αA | Skin | Hunan, China | Negative | MC2 |
| WH063 | αA | CSF | Zhejang, China | Negative | MC2 |
| WH064 | αA | CSF | Hebei, China | Negative | MC2 |
| WH065 | αA | Sputum | Shanghai, China | Negative | MC2 |
| WH066 | αA | CSF | Anhui, China | Negative | MC2 |
| WH067 | αA | CSF | Guangdong, China | Negative | MC2 |
| WH068 | αA | CSF | Shanghai, China | Negative | MC2 |
| WH069 | αA | CSF | Shanghai, China | Negative | MC12 |
| WH070 | αA | CSF | Shanghai, China | Negative | MC2 |
| WH071 | αA | CSF | Shanghai, China | Negative | MC12 |
| WH072 | αA | CSF | Guangdong, China | Negative | MC2 |
| WH073 | αA | CSF | Shanghai, China | Negative | MC2 |
| WH075 | αA | CSF | Beijing, China | Negative | MC2 |
| WH076 | αA | CSF | Guangdong, China | Negative | MC2 |
| WH077 | αA | CSF | Shanghai, China | Negative | MC2 |
| WH078 | αA | CSF | Hebei, China | Negative | MC2 |
| WH079 | αA | CSF | Jiangsu, China | Negative | MC2 |
| WH080 | αA | CSF | Jiangsu, China | Negative | MC2 |
| WH081 | αA | CSF | Zhejang, China | Negative | MC2 |
| WH082 | αA | CSF | Guangdong, China | Negative | MC2 |
| WH083 | αA | Blood | Guangdong, China | Negative | MC2 |
| WH086 | αA | CSF | Shanghai, China | Negative | MC2 |
| WH087 | αA | CSF | Jiangsu, China | Negative | MC2 |
| WH088 | αA | CSF | Zhejang, China | Negative | MC2 |
| WH089 | αA | CSF | Guangdong, China | Negative | MC2 |
| WH090 | αA | CSF | Shanghai, China | Negative | MC2 |
| WH091 | αA | CSF | Guangdong, China | Negative | MC2 |
| WH092 | αA | Urine | Hebei, China | Negative | MC2 |
| WH093 | αA | CSF | Shandong, China | Negative | MC2 |
| WH094 | αA | CSF | Guangdong, China | Negative | MC2 |
| WH095 | αA | Urine | Shanghai, China | Negative | MC2 |
| WH096 | αA | CSF | Hubei, China | Negative | MC2 |
| WH098 | αA | CSF | Shanghai, China | Positive | MC2 |
| WH099 | αA | CSF | Jiangsu, China | Negative | MC12 |
| WH101 | αA | CSF | Zhejang, China | Negative | MC2 |
| WH102 | αA | CSF | Shanghai, China | Negative | MC2 |
| WH103 | αA | Lung | Shanghai, China | Negative | MC2 |
| WH104 | αA | CSF | Zhejang, China | Negative | MC2 |
| WH105 | αA | CSF | Shanghai, China | Negative | MC2 |
| WH106 | αA | CSF | Jiangsu, China | Negative | MC2 |
| WH107 | αA | Skin | Sichuan, China | Negative | None |
| WH108 | αA | CSF | Shanghai, China | Negative | MC2 |
| WH113 | αA | CSF | Anhui, China | Negative | MC2 |
| WH114 | αA | CSF | Zhejang, China | Negative | MC2 |
| WH115 | αA | CSF | Jiangsu, China | Negative | MC2 |
| WH117 | αA | CSF | Hebei, China | Negative | MC2 |
| WH118 | αA | CSF | Shanghai, China | Negative | MC2 |
| WH119 | αA | CSF | Zhejang, China | Negative | MC2 |
| WH120 | αA | CSF | Sichuan, China | Negative | MC2 |
| WH121 | αA | Blood | Guangdong, China | Positive | MC2 |
| WH122 | αA | CSF | Guangdong, China | Positive | MC2 |
| WH123 | αA | CSF | Guangdong, China | Positive | None |
| WH124 | αA | CSF | Guangdong, China | Positive | MC2 |
| WH125 | αA | CSF | Guangdong, China | Positive | MC2 |
| WH126 | αA | CSF | Guangdong, China | Positive | MC2 |
| WH127 | αA | CSF | Guangdong, China | Positive | MC12 |
| WH128 | αA | CSF | Guangdong, China | Positive | None |
| WH129 | αA | CSF | Guangdong, China | Positive | MC12 |
| WH130 | αA | Blood | Guangdong, China | Positive | MC2 |
| WH131 | αA | CSF | Guangdong, China | Positive | MC2 |
| WH132 | αA | CSF | Guangdong, China | Positive | MC3 |
