# Supplementary material for: Resistance of Asian Cryptococcus neoformans Serotype A Is Confined to Few Microsatellite Genotypes
Source: PLoS One. 2012 Mar 13;7(3):e32868. doi: 10.1371/journal.pone.0032868 (PMC3302784; doi:10.1371/journal.pone.0032868)
Supplement: Table S2 — Environmental Cryptococcus neoformans var. grubii isolates from Thailand and Japan. (DOC) [file pone.0032868.s002.doc]

**Table S2. Environmental *Cryptococcus* *neoformans* var. *grubii* isolates from Thailand and Japan**

| Isolate | Mating/Serotype | Source | Geographic origin | MCs |
| --- | --- | --- | --- | --- |
| 34 | αA | avian dropping | Chiang Mai, Thailand | MC2 |
| 40 | αA | avian dropping | Chiang Mai, Thailand | MC2 |
| 44 | αA | avian dropping | Chiang Mai, Thailand | MC8 |
| 109A | αA | avian dropping | Chiang Mai, Thailand | MC8 |
| 109C | αA | avian dropping | Chiang Mai, Thailand | MC8 |
| 110A | αA | avian dropping | Chiang Mai, Thailand | MC15 |
| 110E | αA | avian dropping | Chiang Mai, Thailand | MC8 |
| 130C | αA | avian dropping | Chiang Mai, Thailand | MC8 |
| 130D | αA | avian dropping | Chiang Mai, Thailand | MC8 |
| 189E | αA | avian dropping | Chiang Mai, Thailand | MC2 |
| 2551-07 CM | αA | avian dropping | Chiang Mai, Thailand | MC8 |
| 26A | αA | avian dropping | Chiang Mai, Thailand | None |
| 96B | αA | avian dropping | Chiang Mai, Thailand | MC8 |
| B2 | αA | avian dropping | Chiang Mai, Thailand | MC8 |
| D1 | αA | dove dropping | Chiang Mai, Thailand | MC8 |
| D12 | αA | dove dropping | Chiang Mai, Thailand | MC8 |
| D14 | αA | dove dropping | Chiang Mai, Thailand | MC8 |
| D15 | αA | dove dropping | Chiang Mai, Thailand | MC12 |
| D16 | αA | dove dropping | Chiang Mai, Thailand | MC2 |
| D17 | αA | dove dropping | Chiang Mai, Thailand | MC12 |
| D18 | αA | dove dropping | Chiang Mai, Thailand | MC8 |
| D19 | αA | dove dropping | Chiang Mai, Thailand | MC12 |
| D2 | αA | dove dropping | Chiang Mai, Thailand | MC8 |
| D21 | αA | dove dropping | Chiang Mai, Thailand | MC8 |
| D22 | αA | dove dropping | Chiang Mai, Thailand | MC8 |
| D26 | αA | dove dropping | Chiang Mai, Thailand | MC8 |
| D27 | αA | dove dropping | Chiang Mai, Thailand | MC8 |
| D28 | αA | dove dropping | Chiang Mai, Thailand | MC8 |
| D31 | αA | dove dropping | Chiang Mai, Thailand | MC8 |
| D33 | αA | dove dropping | Chiang Mai, Thailand | MC8 |
| D34 | αA | dove dropping | Chiang Mai, Thailand | MC8 |
| D35 | αA | dove dropping | Chiang Mai, Thailand | MC8 |
| D36 | αA | dove dropping | Chiang Mai, Thailand | MC8 |
| D4 | αA | dove dropping | Chiang Mai, Thailand | MC2 |
| D41 | αA | dove dropping | Chiang Mai, Thailand | MC8 |
| D42 | αA | dove dropping | Chiang Mai, Thailand | MC8 |
| D43 | αA | dove dropping | Chiang Mai, Thailand | MC8 |
| D44 | αA | dove dropping | Chiang Mai, Thailand | MC2 |
| D45 | αA | dove dropping | Chiang Mai, Thailand | MC8 |
| D46 | αA | dove dropping | Chiang Mai, Thailand | MC8 |
| D6 | αA | dove dropping | Chiang Mai, Thailand | MC2 |
| D64 | αA | dove dropping | Chiang Mai, Thailand | MC8 |
| D69 | αA | dove dropping | Chiang Mai, Thailand | MC8 |
| D71 | αA | dove dropping | Chiang Mai, Thailand | MC8 |
| D73 | αA | dove dropping | Chiang Mai, Thailand | MC8 |
| D76 | αA | dove dropping | Chiang Mai, Thailand | MC8 |
| D9 | αA | dove dropping | Chiang Mai, Thailand | MC8 |
| PG1 | αA | pigeon dropping | Chiang Mai, Thailand | MC8 |
| PG2 | αA | pigeon dropping | Chiang Mai, Thailand | MC8 |
| PG21 | αA | pigeon dropping | Chiang Mai, Thailand | MC8 |
| PG26 | αA | pigeon dropping | Chiang Mai, Thailand | MC8 |
| PG3 | αA | pigeon dropping | Chiang Mai, Thailand | MC8 |
| PG32 | αA | pigeon dropping | Chiang Mai, Thailand | MC8 |
| PG37 | αA | pigeon dropping | Chiang Mai, Thailand | MC2 |
| PG46 | αA | pigeon dropping | Chiang Mai, Thailand | MC8 |
| 110C | αA | avian dropping | Chiang Mai, Thailand | MC8 |
| 110D | αA | avian dropping | Chiang Mai, Thailand | MC8 |
| D3 | αA | dove dropping | Chiang Mai, Thailand | MC8 |
| 9211 | αA | avian dropping | Tokyo, Japan | MC2 |
| 9212 | αA | avian dropping | Tokyo, Japan | MC16 |
| 9254 | αA | avian dropping | Tokyo, Japan | MC16 |
| 9256 | αA | avian dropping | Tokyo, Japan | None |
| 9257 | αA | avian dropping | Tokyo, Japan | None |
| 9258 | αA | avian dropping | Tokyo, Japan | MC2 |
| 9259 | αA | avian dropping | Tokyo, Japan | MC16 |
| 9260 | αA | avian dropping | Tokyo, Japan | MC2 |
| 9261 | αA | avian dropping | Tokyo, Japan | MC16 |
