# Supplementary material for: Resistance of Asian Cryptococcus neoformans Serotype A Is Confined to Few Microsatellite Genotypes
Source: PLoS One. 2012 Mar 13;7(3):e32868. doi: 10.1371/journal.pone.0032868 (PMC3302784; doi:10.1371/journal.pone.0032868)
Supplement: Table S3 — The MIC range, MIC50, MIC90, and geometric mean for clinical isolates of C. neoformans in each country. (DOC) [file pone.0032868.s003.doc]

**Table S3. The MIC range, MIC_50_, MIC_90_, and geometric mean for clinical isolates of *C*. *neoformans*** in each country

| Isolates | Antifungal agent | MIC | | | |
| --- | --- | --- | --- | --- | --- |
|  |  | Range | MIC_50_ | Geometric Mean | MIC_90_ |
| Chinese *C*. *neoformans* isolates (*n* = 115) | Amphotericine B | 0.063 - 1 | 0.25 | 0.263 | 0.5 |
|  | 5-Flucytosine | 0.25 - 32 | 4 | 3.054 | 8 |
|  | Fluconazole | 0.125 - 16 | 2 | 2.088 | 4 |
|  | Itraconazole | <0.016 - 0.25 | 0.125 | 0.061 | 0.25 |
|  | Voriconazole | <0.016 - 0.25 | 0.031 | 0.033 | 0.125 |
|  | Posaconazole | <0.016 - 0.5 | 0.063 | 0.054 | 0.125 |
|  | Isavuconazole | <0.016 - 0.125 | 0.031 | 0.020 | 0.063 |
|  |  |  |  |  |  |
| Japanese *C*. *neoformans* isolates (*n* = 28) | Amphotericine B | 0.063 - 0.5 | 0.5 | 0.385 | 0.5 |
|  | 5-Flucytosine | 0.5 - 8 | 4 | 3.075 | 8 |
|  | Fluconazole | 0.25 - 8 | 2 | 2.098 | 4 |
|  | Itraconazole | 0.031 - 0.25 | 0.063 | 0.069 | 0.25 |
|  | Voriconazole | <0.016 - 0.25 | 0.063 | 0.082 | 0.25 |
|  | Posaconazole | 0.031 - 0.125 | 0.063 | 0.074 | 0.125 |
|  | Isavuconazole | <0.016 - 0.125 | 0.031 | 0.031 | 0.063 |
|  |  |  |  |  |  |
| Indian *C*. *neoformans* isolates (*n* = 61) | Amphotericine B | 0.125 - 0.5 | 0.25 | 0.197 | 0.25 |
|  | 5-Flucytosine | 0.5 - 16 | 4 | 3.490 | 8 |
|  | Fluconazole | 0.25 - 16 | 2 | 2.597 | 4 |
|  | Itraconazole | <0.016 - 0.25 | 0.063 | 0.048 | 0.125 |
|  | Voriconazole | <0.016 - 0.125 | 0.031 | 0.034 | 0.063 |
|  | Posaconazole | <0.016 - 0.25 | 0.063 | 0.054 | 0.125 |
|  | Isavuconazole | <0.016 - 0.125 | 0.008 | 0.019 | 0.063 |
|  |  |  |  |  |  |
| Indonesian *C*. *neoformans* isolates (*n* = 40) | Amphotericine B | 0.063 - 0.5 | 0.125 | 0.180 | 0.5 |
|  | 5-Flucytosine | 0.063 - >64 | 4 | 8.284 | 128 |
|  | Fluconazole | 0.5 - 16 | 4 | 3.482 | 16 |
|  | Itraconazole | <0.016 - 0.5 | 0.125 | 0.102 | 0.25 |
|  | Voriconazole | <0.016 - 0.25 | 0.063 | 0.061 | 0.125 |
|  | Posaconazole | <0.016 - 0.25 | 0.125 | 0.093 | 0.25 |
|  | Isavuconazole | <0.016 - 0.125 | 0.031 | 0.031 | 0.063 |
|  |  |  |  |  |  |
| Thai *C*. *neoformans* isolates (*n* = 167) | Amphotericine B | 0.063 - 1 | 0.25 | 0.259 | 0.5 |
|  | 5-Flucytosine | 0.5 - >64 | 4 | 3.391 | 8 |
|  | Fluconazole | 0.125 - 32 | 2 | 2.199 | 4 |
|  | Itraconazole | <0.016 - 0.25 | 0.063 | 0.052 | 0.125 |
|  | Voriconazole | <0.016 - 0.5 | 0.063 | 0.060 | 0.125 |
|  | Posaconazole | <0.016- 0.25 | 0.063 | 0.053 | 0.125 |
|  | Isavuconazole | <0.016 - 0.125 | 0.008 | 0.017 | 0.063 |
|  |  |  |  |  |  |
|  |  |  |  |  |  |
|  |  |  |  |  |  |
| Kuwait *C*. *neoformans* isolates (*n* = 10) | Amphotericine B | 0.125 - 0.25 | 0.125 | 0.154 | 0.25 |
|  | 5-Flucytosine | 1 - 4 | 2 | 2.297 | 4 |
|  | Fluconazole | 1 - 4 | 2 | 2.000 | 4 |
|  | Itraconazole | <0.016 - 0.25 | 0.063 | 0.063 | 0.125 |
|  | Voriconazole | <0.016 - 0.125 | 0.031 | 0.041 | 0.063 |
|  | Posaconazole | <0.016 - 0.125 | 0.063 | 0.055 | 0.125 |
|  | Isavuconazole | <0.016 - 0.125 | 0.031 | 0.029 | 0.125 |
|  |  |  |  |  |  |
| Qatar *C*. *neoformans* isolates (*n* = 5) | Amphotericine B | 0.25 - 0.5 | 0.5 | 0.379 | 0.5 |
|  | 5-Flucytosine | 4 - 8 | 8 | 6.063 | 8 |
|  | Fluconazole | 4 - 8 | 4 | 4.595 | 8 |
|  | Itraconazole | 0.063 - 0.25 | 0.25 | 0.144 | 0.25 |
|  | Voriconazole | 0.063 - 0.125 | 0.125 | 0.095 | 0.125 |
|  | Posaconazole | 0.125 - 0.25 | 0.125 | 0.144 | 0.25 |
|  | Isavuconazole | 0.063 - 0.125 | 0.063 | 0.083 | 0.125 |
