# Supplementary material for: Resistance of Asian Cryptococcus neoformans Serotype A Is Confined to Few Microsatellite Genotypes
Source: PLoS One. 2012 Mar 13;7(3):e32868. doi: 10.1371/journal.pone.0032868 (PMC3302784; doi:10.1371/journal.pone.0032868)
Supplement: Table S4 — The MIC range, MIC50, MIC90, and geometric mean for the different microsatellite complexes (MCs) of C. neoformans. (DOC) [file pone.0032868.s004.doc]

**Table S4. The MIC range, MIC_50_, MIC_90_, and geometric mean for the different microsatellite complexes (MCs) of *C. neoformans***

| Isolates | Antifungal agent | MIC | | | |
| --- | --- | --- | --- | --- | --- |
|  |  | Range | MIC_50_ | Geometric Mean | MIC_90_ |
| *C*. *neoformans* MC1 (*n* = 14) | Amphotericine B | 0.125 - 0.25 | 0.25 | 0.205 | 0.25 |
|  | 5-Flucytosine | 4 - 16 | 8 | 7.614 | 16 |
|  | Fluconazole | 1 - 8 | 2 | 2.692 | 8 |
|  | Itraconazole | <0.016 - 0.25 | 0.063 | 0.051 | 0.125 |
|  | Voriconazole | <0.016 - 0.125 | 0.063 | 0.044 | 0.063 |
|  | Posaconazole | <0.016 - 0.125 | 0.063 | 0.054 | 0.125 |
|  | Isavuconazole | <0.016 - 0.125 | 0.031 | 0.031 | 0.063 |
|  |  |  |  |  |  |
| *C*. *neoformans* MC2 (*n* = 151) | Amphotericine B | 0.063 - 1 | 0.25 | 0.282 | 0.5 |
|  | 5-Flucytosine | 0.25 - 32 | 4 | 3.479 | 8 |
|  | Fluconazole | 0.125 - 16 | 2 | 2.365 | 4 |
|  | Itraconazole | <0.016 - 0.5 | 0.125 | 0.068 | 0.25 |
|  | Voriconazole | <0.016 - 0.25 | 0.063 | 0.042 | 0.125 |
|  | Posaconazole | <0.016 - 0.5 | 0.063 | 0.063 | 0.125 |
|  | Isavuconazole | <0.016 - 0.125 | 0.031 | 0.027 | 0.063 |
|  |  |  |  |  |  |
|  |  |  |  |  |  |
| *C*. *neoformans* MC3 (*n* = 42) | Amphotericine B | 0.063 - 0.5 | 0.25 | 0.195 | 0.25 |
|  | 5-Flucytosine | 0.5 - >64 | 4 | 4.203 | 8 |
|  | Fluconazole | 0.25 - 16 | 2 | 2.923 | 8 |
|  | Itraconazole | <0.016 - 0.25 | 0.063 | 0.053 | 0.125 |
|  | Voriconazole | <0.016 - 0.25 | 0.031 | 0.036 | 0.063 |
|  | Posaconazole | <0.016 - 0.25 | 0.063 | 0.059 | 0.125 |
|  | Isavuconazole | <0.016 - 0.125 | 0.016 | 0.023 | 0.063 |
|  |  |  |  |  |  |
| *C*. *neoformans* MC8 (*n* = 206) | Amphotericine B | 0.125 - 1 | 0.25 | 0.244 | 0.5 |
|  | 5-Flucytosine | 0.063 - >64 | 4 | 2.838 | 4 |
|  | Fluconazole | 0.125 - 32 | 2 | 2.020 | 4 |
|  | Itraconazole | <0.016 - 0.5 | 0.063 | 0.059 | 0.125 |
|  | Voriconazole | <0.016 - 0.5 | 0.063 | 0.054 | 0.125 |
|  | Posaconazole | <0.016 - 0.25 | 0.063 | 0.057 | 0.125 |
|  | Isavuconazole | <0.016 - 0.125 | 0.031 | 0.024 | 0.063 |
|  |  |  |  |  |  |
| *C*. *neoformans* MC12 (*n* = 13) | Amphotericine B | 0.125 - 0.5 | 0.25 | 0.264 | 0.5 |
|  | 5-Flucytosine | 1- 4 | 2 | 2.347 | 4 |
|  | Fluconazole | 0.25 - 8 | 2 | 1.896 | 8 |
|  | Itraconazole | <0.016 - 0.25 | 0.125 | 0.074 | 0.125 |
|  | Voriconazole | <0.016 - 0.125 | 0.063 | 0.054 | 0.125 |
|  | Posaconazole | <0.016 - 0.125 | 0.063 | 0.059 | 0.125 |
|  | Isavuconazole | <0.016 - 0.063 | 0.031 | 0.026 | 0.063 |
|  |  |  |  |  |  |
|  |  |  |  |  |  |
| *C*. *neoformans* MC15 (*n* = 11) | Amphotericine B | 0.125 - 0.25 | 0.25 | 0.207 | 0.25 |
|  | 5-Flucytosine | 2- 4 | 2 | 2.416 | 4 |
|  | Fluconazole | 2 - 4 | 4 | 3.109 | 4 |
|  | Itraconazole | 0.031 - 0.125 | 0.063 | 0.062 | 0.125 |
|  | Voriconazole | 0.031 - 0.063 | 0.031 | 0.043 | 0.063 |
|  | Posaconazole | 0.031 - 0.25 | 0.063 | 0.086 | 0.125 |
|  | Isavuconazole | <0.016 - 0.063 | 0.031 | 0.033 | 0.063 |
|  |  |  |  |  |  |
| *C*. *neoformans* MC16 (*n* = 21) | Amphotericine B | 0.25 - 0.5 | 0.5 | 0.397 | 0.5 |
|  | 5-Flucytosine | 1 - 8 | 4 | 3.391 | 4 |
|  | Fluconazole | 0.25 - 8 | 2 | 2.208 | 4 |
|  | Itraconazole | 0.031 - 0.25 | 0.063 | 0.084 | 0.25 |
|  | Voriconazole | <0.016 - 0.25 | 0.125 | 0.090 | 0.25 |
|  | Posaconazole | 0.031 - 0.25 | 0.063 | 0.082 | 0.125 |
|  | Isavuconazole | <0.016 - 0.125 | 0.031 | 0.038 | 0.063 |
|  |  |  |  |  |  |
| *C*. *neoformans* MC17 (*n* = 15) | Amphotericine B | 0.125 - 0.5 | 0.25 | 0.228 | 0.5 |
|  | 5-Flucytosine | 1 - >64 | 8 | 12.7 | >64 |
|  | Fluconazole | 1 - 16 | 4 | 3.482 | 16 |
|  | Itraconazole | <0.016 - 0.25 | 0.125 | 0.099 | 0.25 |
|  | Voriconazole | 0.031 - 0.25 | 0.063 | 0.079 | 0.25 |
|  | Posaconazole | 0.063 - 0.25 | 0.125 | 0.109 | 0.25 |
|  | Isavuconazole | <0.016- 0.125 | 0.031 | 0.039 | 0.063 |
